# Complete mitochondrial genome of Sichuan’s population of *Aphis aurantii* (Hemiptera: Aphididae)

**DOI:** 10.1080/23802359.2020.1715303

**Published:** 2020-05-20

**Authors:** Deqiang Pu, Chao Liu, Hongling Liu, Zhi-Teng Chen, Xinglong Wu, Kejun Xiao, Xi Luo, Jianhui Mao, Qiong Huang

**Affiliations:** a Key Laboratory of Integrated Pest Management of Southwest Crops, Institute of Plant Protection, Sichuan Academy of Agricultural Sciences, Chengdu, China; b College of Forestry, Sichuan Agricultural University, Wenjiang, China; c School of Grain Science and Technology, Jiangsu University of Science and Technology, Zhenjiang, China

**Keywords:** Mitochondrial genome, *Aphis aurantii*, phylogeny

## Abstract

The complete mitochondrial genome of the black citrus aphid from Sichuan Province of China, *Aphis aurantii*, was sequenced and analyzed. The mitochondrial genome was a double strand, circular molecule with 15,296 bp and an A + T content of 83.5%, comprising 13 PCGs, 22 tRNA genes, and two rRNA genes. Gene arrangement was conserved in the mitogenome of *A. aurantii*. A 631-bp long control region was found, with a high A + T content of 82.6%. All PCGs used standard ATN start codons and most PCGs ended with complete TAA stop codons. The phylogenetic analysis supported that *A. aurantii* was closely related to other five congeners of the genus *Aphis*.

*Aphis aurantii* (Boyer de Fonscolombe) (Hemiptera: Aphididae), also known as the black citrus aphid, is a worldwide transmission of citrus tristeza virus (CTV) and major pest of citrus (Wang and Tsai 2001). Genetic biodiversity of this pest in China is still unclear, which can be resolved by sequencing more genetic data of different geographical populations. To provide more genetic data for *A. aurantii*, we sequenced the complete mitogenome of Sichuan’s population of *A. aurantii* using Illumina Hiseq 4000 (Shanghai BIOZERON Co., Ltd). The specimens of *A. aurantii* were collected from Chenjiaba Town, Beichuan County, Sichuan Province, China (31°55′53.99″N, 104°35′16.13″E) in April 2019. All specimens and isolated DNA samples were stored in the Insect Collection of Sichuan Academy of Agricultural Sciences (ICSAAS, No. ICSAAS-HEM-APHI1), Chengdu, China. The mitogenome sequence was deposited in GenBank with the accession number MN871977.

The complete mitogenome of *A. aurantii* has a length of 15,296 bp and contains an A + T content of 83.5% (A: 44.8%, T: 38.7%, C: 10.6%, G: 5.9%). A total of 37 genes (13 PCGs, 22 tRNA genes, and two rRNA genes), and a non-coding control region were annotated. The control region was located between *rrnS* and *trnIle*, 631 bp in length with an A + T content of 82.6%. In the mitogenome of *A. aurantii*, no gene rearrangement was detected.

All the PCGs started with the standard start codon ATN (ATA, ATT, and ATG); most PCGs terminated with the complete stop codon TAA, whereas *nad4* ended with an incomplete codon T—. The 22 tRNA genes varied in length from 62 bp to 80 bp, mostly showing clover-leaf structures except for *trnSer1 (AGN)*, the DHU arm of which was completely lost. Two rRNA genes, *rrnL* and *rrnS*, were found in the conserved locations between *trnSer1 (AGN)* and the control region. The *rrnL* gene was 1275 bp in length with an A + T content of 84.6%. The *rrnS* gene was 769 bp in length with an A + T content of 84.3%. There were 73 overlapped nucleotides between 14 gene pairs with the longest overlap between *atp6* and *atp8*. A total of 272 intergenic nucleotides were found between 13 gene pairs, with the longest 179-bp intergenic sequence between *trnPhe (F)* and *trnGlu (E)*.

Phylogenetic relationship within Aphididae was reconstructed based on the nucleotide sequences of 13 PCGs. The Bayesian inference (BI) method generated a tree topology identical to that of Wang et al. (2019) (Figure 1). *Aphis aurantii* sequenced in this study was grouped with other five congeners of *Aphis*, which supported the efficiency of mitogenome data in phylogenetic analysis and confirmed the morphological identification of the sequenced specimens. *Cervaphis quercus* was recovered as a relatively basal species to other aphids used in this study. The phylogeny of Aphididae is still unresolved, requiring more molecular data including mitogenome data to solve this problem.

**Figure 1.**
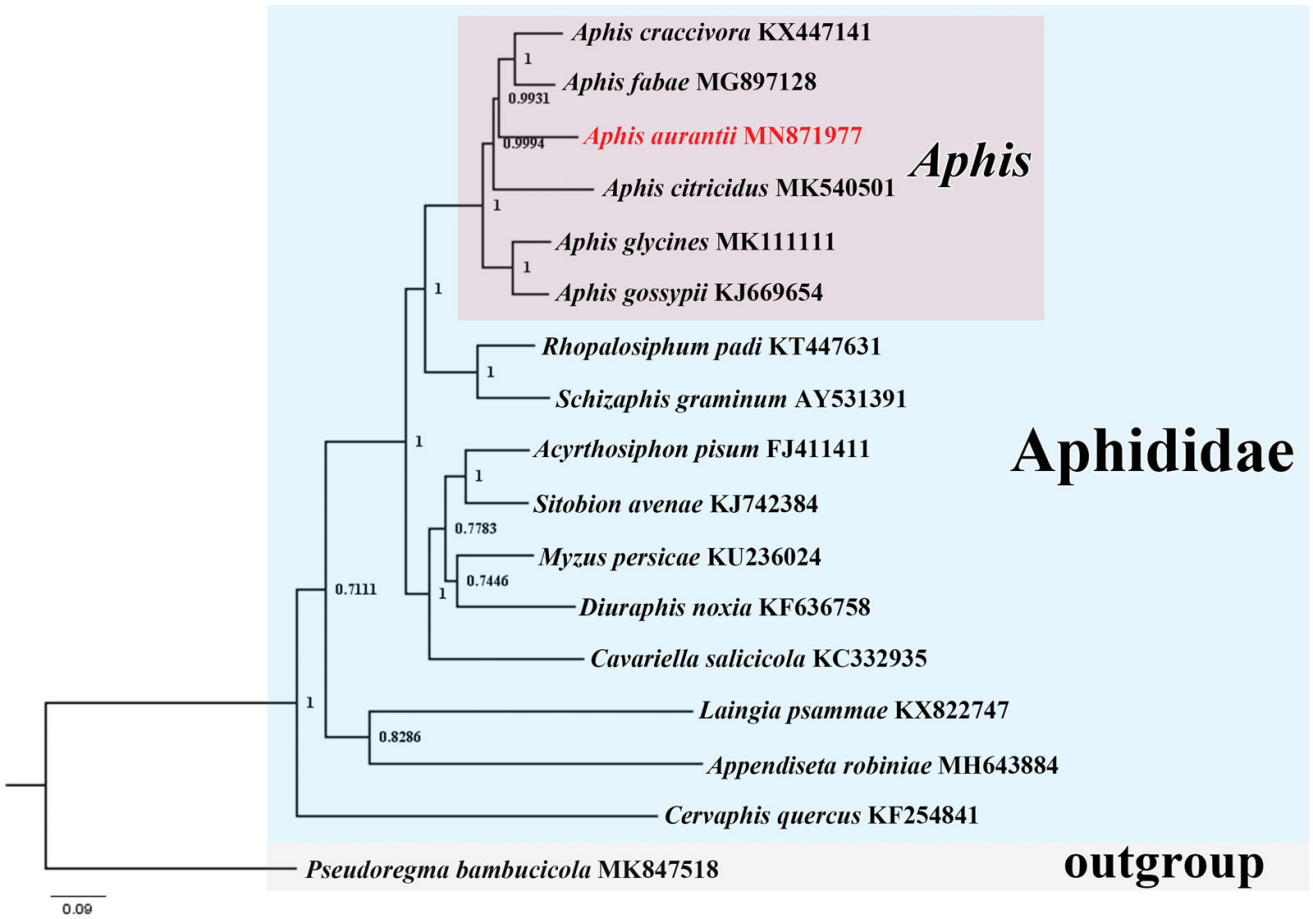
Phylogenetic tree of 16 species of Aphididae. Numbers at the nodes are posterior probabilities. The GenBank accession numbers are indicated after the scientific names. The tree is rooted with *Pseudoregma bambucicola* (MK847518).
